# New Inventions

**Published:** 1896-01

**Authors:** 


### NEW INVENTIONS.

A knee-spreading device made secure to the shaft and to the horse's leg by a strap at the knee, the connection being a spring-bracket.

A combined feed-rack and trough, with means for adjustment of trough and rack at varying heights.

A powder-blower, working somewhat on the principle of hand-bellows.

A hoof-pad, to be fastened between shoe and hoof, having an unyielding rim of a shape and size to fit the foot; with this a felt cushion over the bottom of the foot, the cushion depressed to allow room for frog, and cushion fastened to a series of rands.

A nailless horseshoe of two sections, pointed at toe and made secure by a series of flanges, with a screw-clamping device at the heel.

A means for analytically recording the gait of horses, consisting of an electrical circuit, with wires to each hoof, arranged to work independently of each other, connected with a battery; the whole arranged to work in conjunction with a vehicle.

A bridle, composed of forked sides, with a bit between lower part, the upper part extending upward continued over nose by a nose-band and backward to over cheek. Also arranged on lower part for adjustable curb.

A device for stopping runaway horses, comprising a pair of pivotally connected jaws, carrying knobs at their forward ends to flank the nostrils.

A thermo-cautery, having a carburetter and cauterizing-point in combination with a blowpipe-torch, adapted to be held adjacent to the point.

A device to prevent feather-pulling in fowls, consisting of a single piece of spring wire, the free ends bent outward and inward to form curved hooks and fastened to the bills.

A horse-hitching device, consisting of a pair of tongs, like metal jaws, so arranged by a handle to open and shut as desired.

A thermo-cautery, with a carburetter and an air-forcing device connected with one end, an arm secured to the other end of the carburetter, having a pleurality of air-passages therein.

A toe-weight, consisting of a base-plate fastened to the foot, a threaded stud extending from the face thereof, and a removable screw-weight.

A horseshoe calk sharpener.

An improved horseshoe, composed of two sections and an interposed elastic filling.

A muzzle made in sections, so as to open or shut, and adjusted, whereby the same may be operated.

A letter and message receiver-box, with place on front to write message, and by turning knob the message is placed in box and a blank sheet takes its place to receive others. Also plate to designate the hour of return to office.

An improved artery-forceps, so arranged as to prevent longitudinal movement after locking of the spring.

An interfering-pad, to be adjusted between the shoe and hoof by a tongue, and held in place by a strap around the hoof, partly composed of rubber-tubing.

A currycomb, adapted to bind the shedder rigidly against the comb-bars.

A harness-padhook for fastening pads to harness on various parts of body.

A cushioned horseshoe, partly metal, which is irregularly recessed on the plantar surface to receive irregular shaped particles of rubber, so as to insure a firm attachment between the two parts.

